# Discovering strengths in patients with medically unexplained symptoms – a focus group study with general practitioners

**DOI:** 10.1080/02813432.2022.2139345

**Published:** 2022-11-08

**Authors:** Ingjerd Helene Jøssang, Aase Aamland, Stefan Hjörleifsson

**Affiliations:** a Department of global public health and primary care, University of Bergen, Norway; b Research unit for general practice, NORCE Norwegian Research Centre, Bergen, Norway

**Keywords:** MUS, medically unexplained symptoms, strengths, epistemic disadvantage, focus groups, co-subject

## Abstract

**Background:**

When patients suffer medically unexplained symptoms, consultations can be difficult and frustrating for both patient and GP. Acknowledging the patient as a co-subject can be particularly important when the symptoms remain unexplained. One way of seeing the patient as a co-subject is by recognizing any among their strong sides.

**Objectives:**

To explore GPs’ experiences with discovering strengths in their patients with medically unexplained symptoms and elicit GPs’ reflections on how this might be useful.

**Methods:**

Four focus-groups with 17 GPs in Norway. Verbatim transcripts from the interviews were analyzed by systematic text condensation.

**Results:**

Recollecting patients’ strengths was quiet challenging to the GPs. Gradually they nevertheless shared a range of examples, and many participants had experienced that knowing patients’ strong sides could make consultations less demanding, and sometimes enable the GP to provide better help. Identifying strengths in patients with unexplained symptoms required a deliberate effort on the GPs’ behalf, and this seemed to be a result of a strong focus on biomedical disease and loss of function.

**Conclusions:**

Acknowledging patients’ strong sides can bolster GPs’ ability to help patients with medically unexplained symptoms. However, the epistemic disadvantage of generalist expertise makes this hard to achieve. It is difficult for GPs to integrate person-centered perspectives with biomedical knowledge due to the privileged position of the latter. This seems to indicate a need for system-level innovations to increase the status of person-centered clinical work.

**Key points**
- MUS is challenging for both patients and GPs mainly because of the incongruence between symptoms and the dominating biomedical model.
- GPs’ focus on pathology and loss of function can prevent them from discovering patients’ strengths.
- Awareness of patients’ strengths can make consultations less demanding for GPs and enable them to provide better help.
- A conscious effort is needed to discover patients’ strengths.

## Introduction

General practitioners (GPs) meet many patients with persistent and disabling symptoms without corresponding objective findings. Such symptoms do not fit with the traditional biomedical disease model. ‘Medically unexplained symptoms’ (MUS) is one of the notions used for such conditions [1]. Conditions like chronic fatigue syndrome, irritable bowel syndrome and fibromyalgia are among the most well-known MUS entities, while musculoskeletal pain, tiredness, gastrointestinal complaints, dizziness and different sensory symptoms are often also ‘medically unexplained’. GPs play a key role in the follow-up and management of patients with MUS. Although ascertaining the prevalence of MUS is notoriously difficult, we know that the prevalence in general practice is high [2]. Depending on the definition of MUS, studies have suggested that a range between 3% and more than 2/3 of all consultations in general practice involve MUS [3–5]. MUS also cause big costs in society due to for instance health care use and sick leave [6,7].

Many patients with MUS report less satisfaction with consultations with their GP than patients with traditional biomedical diseases [8]. Patients experience that their symptoms are not credible and GPs experience that their medical knowledge is not relevant or useful [9,10]. In the absence of commonly accepted explanatory models and limited efficacy of therapeutic tools, the patient and the GP may feel stuck, helpless and unworthy [4,11], and many GPs hold negative attitudes towards MUS [12,13]. Newly graduated GPs often experience discrepancy between the ideal they learned while studying diseases with objective findings and biological origin, and the reality they meet with patients who suffer from MUS [14]. With experience it may get easier to accept uncertainty and manage meetings with MUS patients [15,16]. Although Aamland et al. found that many GPs have developed helpful strategies in seeing patients with MUS [17], their study did not reveal strategies having to do with acknowledging patients’ resources or strengths which is the focus of our study. A meta-synthesis suggests that the challenges GPs experience can be interpreted as the result of an epistemological incongruence between the traditional biomedical model and the reality the GPs meet in patients with MUS [4].

The current study was performed to supplement existing research on how GPs manage their patients with MUS by focusing on subjectivity and patient resources. It is well known that in general practice focus on patients’ resources can contribute to better health as a supplement to a biomedical focus on pathology and disease [18,19]. The biomedical model has been criticized for excluding the patients personal or subjective standpoint [20]. Kirkengen et al. describe MUS as an anomaly within the biomedical model [21], calling for a non-reductionistic understanding of what it is to be a person. In situations where current medical knowledge has significant shortcomings, knowledge about patients’ resources may be particularly relevant for GPs’ ability to provide support. More specifically, acknowledging the patient as a co-subject can be particularly important when the patient’s symptoms remain unexplained for the GP [21].

Antonovsky’s work on salutogenesis recommends a focus on the human need for ‘sence of coherence’ [22]. Rather than focusing on pathology and disease, focus on supporting the patients’ possibility to experience meaning and coherence in life can enhance the patients’ use of their own resources. Building on Antonovsky’s theories, Malterud and Hollnagel have claimed that self-assessed health resources may be as important as conventional risk factors in preventive health care [23].

The patients experience of respect and acknowledgement from the doctor can be a decisive prerequisite for good health care [24]. A Danish study showed that patients with MUS had a need for existential acknowledgement, while the experience of being met with disrespect and distrust connects with development of increased stress and focus on symptoms [25]. Further, patients with MUS more often than other patients wanted emotional support and acknowledgement from the GP [26]. Patients are not only objects, but also persons, and it is likely to think that when dealing with patients with MUS it is of big importance that the GP takes this into consideration.

One way of seeing the patient as a co-subject is by acknowledging any among their strong sides. We therefore aimed to collect GPs’ accounts of episodes where they had discovered strengths in their patients that they did not know from before. Further, we wanted to explore the GPs’ reflections on how this knowledge might be useful. In this study we understand ‘strengths’ widely, so as to include personal resources, personality traits, skills involved in hobbies, etc.

## Method

### Recruitment and sample

We performed an interview study with four focus groups in Norway in spring 2018. All 17 participants were GPs (five women and 12 men), and the number of participants in each group varied from three to six. We recruited peer groups of GPs who meet on a regular basis as part of the mandatory program for recertification. Each group was recruited by email communication with one of its members identified by convenience within our extended professional network. The age of the participants ranged from 35 to 74 years, median age 42 years, and their working experience ranged from two to 44 years, median nine years. The GPs worked both in rural and urban areas.

### Data collection

The focus group design was chosen to take advantage of communicative interactions between the participants when sharing their experiences [27]. Each interview lasted approximately 90 min, and the moderator used an interview guide (included in Appendix 1). The topic and aim were presented to the participants via email before the interview, and the participants were asked to think of one or two examples of strengths in their patients with MUS. At the start of the interview, the participants were handed a sheet of paper with the agenda, presented in Box 1. All three researchers are experienced general practitioners and part time researchers. The first author who is a ph.d.-candidate moderated all four groups, while one of the other authors co-moderated each of the first three groups. The interviews were audio recorded. Ethical approval was obtained from the Regional Committee for Medical and Health Research Ethics.

## Analysis

Verbatim transcripts from the interviews were analyzed in an iterative process by use of systematic text condensation [28]. This is a thematic cross-case analysis comprising four steps. The first step is reading all the material to get an overview and identify preliminary themes, while bracketing preconceptions. The second step is developing code groups from the preliminary themes by identifying meaning units that represent the different aspects of reflections that came up. The third step is developing subgroups that represent important aspects of each code group and condense the content of these subgroups and further on finding quotes, which further illuminate the content. The fourth step is synthesizing the condensates from step three, reconstructing each category. We did not use any software program for analysis, and all authors were involved in each step in the analysis process.

Initially we planned for three focus groups, but after the first interview we realized that more data was necessary due to the low number of participants in the first group, and the data in this group not being as rich as we aimed for. We used the method of information power to evaluate the data [29]. The evaluation of information power depends on five aspects of the data; the aim of the study, sample specificity, use of established theory, quality of the dialogue and analysis strategy. By evaluating each of these aspects we found that the information power was sufficient after four focus group interviews.

Perspectives about the patient as a co-subject with a lived life and a lived body that marks the patients’ experience and understanding of sickness and health contributed to focus our analysis [20,30], though not as a template framework [31].

## Results

In each focus group, the participants initially found it challenging to come up with examples. The analysis revealed that discovering strengths does not seem to come easily for the participants, and they sought to come up with reasons why it was difficult. After reflecting on why it was challenging, they also described the group discussion itself as an eye-opener. As the conversation progressed, the participants shared many stories about different strong sides in their MUS patients. From the participants’ accounts, it was evident that a deliberate effort would often be necessary for the GP to discover these strengths. The participants had experienced that knowledge about the patients’ strong sides could make the consultations less demanding for them and could enable them to provide better help. These findings are elaborated below.

### Difficult for GPs to acknowledge patients’ strengths

Reporting their experiences with discovering strengths was challenging to the GPs. Initially they did not come up with many examples. Gradually, however, they could recollect more examples. In all four focus groups the participants spontaneously reflected on the meaning of ‘strengths’, and some said that although they were sure that patients with MUS have many strong sides, it was hard for them to come up with examples. They were perplexed about why it was so hard for them to recollect strengths during the interviews and why they often did not familiarize themselves more systematically with patients’ strong sides in spite of previously having learnt communication skills for that purpose, and even having experienced the beneficial effect this could have. Several participants suggested that they used to be better at searching for strengths in their patients earlier in their career. They recollected that it usually would have a good effect and pondered why they had abandoned this way of working.

Some expressed embarrassment and concluded that there is probably much the GP does not know about their patients. One explanation could be that in their work there were ample reasons to focus on symptoms and loss of function, leaving little room for acknowledging the patients’ strengths. Reflecting on an example regarding a patient who consulted for documentation for his disability pension, one of the participants concluded thus:

I feel that the patients must convince everyone that they are sick enough. They have to emphasize their weakness to achieve their financial benefits. In these situations, it can be challenging to look for strong sides. *Ben*

Several stated that they experienced the group discussion itself as an eye-opener and that they would look for strengths more often in the future. One participant said they needed to be reminded that it is not all misery and another stated that ideally, we should do this to a larger degree. Some also said that they thought they had a lot more to learn about how to support their patients with MUS.

### Discovering strengths requires awareness

When the participants explained the different strengths they saw, the analysis showed that the participants also then indirectly described how they discovered the strong sides. It appears to depend on a deliberate effort on the GPs behalf to actually make use of the opportunities to find strengths.

Of relevance was the fact that patients with MUS are patients whom the GP follows over a long period of time, often with consultations at regular intervals. With some patients it might feel natural to ask directly what they are able to do despite their ailments. On other occasions, the patient might suddenly start sharing something from their life and it would be up to the GP to take notice of this and recognize its relevance. Several participants said they could be surprised by what sometimes emerged in such situations, even with patients they had known for many years. This could even make the GP embarrassed as they felt they ought to have made the discovery at an earlier point. One participant referred how a patient with fibromyalgia whom he had known for a long time suddenly told him that he had great skills in genealogy. Another experienced participant described her surprise during a consultation with a patient with chronic fatigue syndrome, a patient that she did not get very well along with:

Suddenly she told me that she had started scrapbooking. *Frida*

The participants also shared examples of learning something new about patients when meeting them outside the surgery, e.g. in the public library, at the cinema or at the grocery store. A participant working in a rural area described it like this:

I often bump into him in my own spare time. He plays football, he is in the public library. Wherever something happens, I see him there. And then I think that this is so positive. He is very eager to engage in the local community. *Theodor*

### Strengths come in many shapes

The participants gradually came up with many different types of strong sides they had discovered in their patients with MUS. Although we have sorted the strengths into three main groups below, the diversity among the patients’ strong sides should in itself be considered a main finding.

Firstly, stories from the patients’ everyday life came up throughout each of the interviews. Some involved hobbies and leisure time activities that the patients were able to pursue despite their symptoms. Others would engage in activities in the local community and voluntary work. Some participants shared stories about patients who turned out to have a great sense of humor, and many examples involved patients with MUS showing great support for their families and friends, as in this example regarding a female patient with fibromyalgia whom the participant had known for several years:

One of her strongest sides was that she was a brilliant head of the family. *Ella*

The second theme was the patients’ ability to maintain a positive attitude despite their troubles. Thus, patients would for example prioritize the activities that were important in their lives, and to adjust their energy in a reasonable way. Others would avoid activities that were energy draining, but some examples also involved patients gritting their teeth, pulling themselves together and testing themselves in what they were able to manage in their everyday life. Several participants also highlighted how the patient’s sense of humor could represent the patient’s positive approach in an otherwise difficult consultation:

And then sometimes we are able to laugh our way out of the office. *Agnes*

A third theme was the patients’ fight for recognition for a particular explanation for their symptoms. Some patients would make a great effort to gain acceptance for the explanation they had identified, even if the GP considered this explanation to be far-fetched or simply did not agree. Some would search the Internet for explanations for their condition or seek alternative treatments. One participant said he sometimes felt that the patient could push this so far that it became destructive and unhealthy. However, the participants also acknowledged that this could be regarded as a strength even in situations where they did not agree with the patient:

This demonstrates a coping strategy that helps the patient maintain their self-respect by showing that they know as much as, or even more, than the doctor. *Paul*

### Seeing strengths benefits the GP-patient relation

The participants reflected on how knowledge of patients’ strengths could be useful for their patients and themselves.

According to the participants, one of the reasons why it could be important to be aware of the patients’ strengths was that this could change the way the GP interpreted the patients’ stories and lives. It gave the GP an increased understanding of why things are as they are. One participant said that knowing that the patient can support a family member with severe illness, despite her own challenges, changed the way they communicated and could change the focus in the consultations:

It became a turning point in such a way that it was now possible to meet her on her positive resources. *Frida*

These changes in how they perceived the patient could become a tool for the GP to both understand and provide better for help their patients. It could allow the GP to build a stronger alliance with the patient, and on some occasions enable them to help patients by encouraging them to engage in particular activities in the local community and to use their strengths as a resource for the patients to make changes in their lives.

One example of how it could make the job easier for the GP was when a participant described how discovering a strength in a patient helped her to mobilize tolerance and endurance in a challenging patient-doctor relationship. Another stated that experiencing this positivity in the consultation helped her from feeling pushed up against the corner. Others said it was easier to prevail with the patient when the consultation was focused in a positive direction instead of talking about illness and complaints only. One participant described that knowing about a MUS-patient’s strong interest in singing or something similar could change the atmosphere in the consultation and affect the GP directly in a positive way:

Such experiences give me an energy boost, especially if the relation with that patient is challenging. *Judith*

## Discussion

### Summary of main findings

The GPs in this focus group study reported that identifying a strength in patients with MUS could make consultations less demanding and enable them to provide better help. However, although they could recall many different examples of strong sides in their patients, the analysis indicates that it takes a deliberate effort on behalf of the GP to discover these strengths.

### Findings in relations to other studies

Our theoretical motivation for this study is based on seeing the patient as a co-subject with individual experiences and understanding of sickness and health. Discovering a strength in the patient is one way of seeing and acknowledging the patient as a person. As mentioned above this aligns with Antonovsky’s theory of salutogenesis and sense of coherence, patient-centered medicine and critique of the biomedical paradigm.

Related to this, Van Houwen et al. have found that patients with MUS want to be taken seriously and value a personalized approach in which their GP pays attention to their personal circumstances [32], and they want proper conversations where they are treated as equal partners and where the GP endeavors to establish a genuine contact [33]. Another study shows that also GPs acknowledge the importance of good communication with patients with MUS, and they suggest that communication can be improved by for example communicating in a more patient-centered way [34]. The GPs in our study reported that in their experience, consultations could become less demanding, and they could provide better help when they acknowledged a strength in their patient. Our study thus suggests that in a relationship that may be fraught with a potential for misunderstandings and mutual experience of rejection, acknowledging strengths can enable GPs to connect better with their patients [4].

Salmon et al. found that patients with MUS seek more emotional support than other patients [26]. It seems to us that identifying patients’ strengths can create a better connection between doctors and their patients. Several studies describe that GPs often experience consultations with MUS patients as hard and challenging [13,35–37]. The GPs we interviewed said that discovering a strength like for instance a patient’s strong sense of humor, could change the way they perceived the patient, and thereby make it easier to prevail in a difficult doctor-patient relationship. Related to this, employing laughter and humor during the consultation could also be seen as a way of creating space for emotional support.

Our finding that the patients’ fight for recognition as a coping strategy to maintain self-respect could be regarded as a strong side in the patient can be related to a study by Werner and Malterud [38]. They describe how being a credible patient and maintaining self-esteem and dignity can require hard work for women with chronic pain. Werner and Malterud found that patients often struggled to maintain a balance between being seeen as too weak or too strong, too sick or too healthy, and sought a somatic rather than psychiatric diagnosis. However, our study highlights another aspect of the fight for recognition when the struggle for an acceptable diagnosis carries the cost of being perceived as a difficult patient by the physician. Another aspect of patients’ fight for a diagnosis or explanation is described in a study from Wileman, May and Chew-Graham [35], who saw that GPs could experience patients gaining authority by undermining the opinion of the doctor, or patients calling the GPs’ skills into doubt. Based on our findings, one may hypothesize that acknowledging the patient’s struggle for diagnosis and treatment as a sign of strength might contribute to a more respectful relationship.

Subjective health complaints often lead to absence from work [39]. Some of the participants in our study said that there was little room for looking at strengths when the social security system requires them to focus on symptoms, disabilities, and biomedical findings. Aarseth et al. found that in medical certificates written by GPs in Norway, patients are portrayed as passive carriers of symptoms, and the texts are written in a doctor-orientated rather than patient-centered manner [40]. Indirectly, the social security system may limit the ability of GPs and patients to focus on patients’s strengths and skills.

The finding that our participants initially struggled with finding examples of strong sides in their patients, supports the research of Mjølstad et al. [41] who have investigated what GPs actually know about their patients as persons. They found that GPs were able to describe the personality and occupation of their patients as well as their closest family relations but tended to have less knowledge about their interest and hobbies. The GPs had least knowledge about their social background. In a similar way as in our study, Mjølstad et al. found that some of the GPs reacted with surprise or embarrassment on discovering the gaps in their knowledge about patients they had known for years. They were perplexed about why it was so difficult for them when they at the same time reported that they knew this knowledge could be very useful.

Some of the participants in our study referred to communication methods called ‘key questions’ to reveal knowledge about the patient. Malterud originally developed key questions to invite patients to share their knowledge and experiences [42]. Malterud and Hollnagel subsequently developed specific key questions for eliciting self-assessed health resources in women, based on theoretical perspectives concerning salutogenesis, patient-centeredness and gender studies [42,43]. This way of using communicative action can provide a tool for changing the subject of the conversation from risk and disease to resources and strengths. While the reference to ‘key questions’ among some of the GPs in our study indicates that they may to some extent be familiar with tools for engaging with patient’s strengths, there seems to be a barrier against using such tools. We suggest this might be understood as a consequence of the privileged position of biomedical knowledge. In the following we will seek to interpret our findings in the light of reflection upon action and at an epistemic level.

In a focus group study with experienced GPs, Aamland et al. elicited the GPs’ accounts of reflections on strategies they had found helpful when treating patients with MUPS [17]. Aamland et al. emphasize the importance for clinicians to learn from their encounters with MUS patients with reference to the concept of reflection upon action [44]. In our study, however, we found that reflecting upon their own actions could be challenging to the participants. It may seem that the GPs have a limited vocabulary and skills for reflecting on the person-centered aspects of their own interactions with patients with MUS.

While our study was not designed as an intervention, our interview questions still challenged the GPs to reflect upon the ways in which knowledge of MUS patients’ strengths might be useful. Discovering that it was hard even to remember examples, they also started to discuss why this was so. Such considerations have the potential to initiate a change in the participants’ thinking, which again might influence their clinical practice. In this sense, qualitative research can enable reflection upon action facilitating learning rather than providing neutral descriptions only [45].

Knowledge, including that of researchers, clinicians and patients, is always situated and contested [46]. A previously mentioned analysis has suggested that GPs may experience an incongruence between the traditional biomedical model and their meetings with patients with MUS [4]. While the current study demonstrates that GPs also relate to these patients in ways that are not based on the biomedical model, it also shows it can be challenging for GPs to mobilize these ways of engaging with patients, and the GPs in our study found it hard to articulate their experiences in this regard. The difficulties in identifying patients’ strengths that the GPs in our study experienced can be seen as a case of the epistemic disadvantage of generalist expertise [47], i.e. difficulties in integrating their relation to the patient as a person into the GPs’ understanding of their professional role due to the privileged position of biomedical knowledge. In each of the focus groups, the GPs initially focused on symptoms, loss of function and other negative aspects of MUS when describing their patients. This is compatible with a biomedical approach where doctors are expected to explore and intervene against pathology. Conversely, this focus does not render the patient visible as a person. Even though the biopsychosocial model should be well-known among GPs in Norway, it seems to take a deliberate effort on the GPs’ behalf to avoid operating uniquely within the biomedical paradigm. We suggest that this can be explained by reference to the epistemic disadvantage of generalist expertise.

### Strengths and limitations

Focus groups can reveal what the participants believe that they do or what they choose to report, and do not provide direct data regarding what happens in patient-doctor interactions [48]. However, focus groups are useful for exploring the participants’ reflections based on their own experiences, and the discussion can facilitate new insights among the participants. We did not use purposeful sampling, but we saw that we reached a good distribution of age, years of working experience and work location (rural and urban practices). There was an overweight of male participants, but this study does not analyze the results from a gender perspective. Both the finding that patients’ fight for recognition can be regarded as a strength and the finding that it required a significant amount of work for the participants to come up with examples of patients’ strong sides came as a surprise to us. The extent of unanticipated findings adds to the validity of the study results, in particular when seen in combination with the participants’ reflections on the reasons why identifying strengths in their patients was hard for them. On the other hand, it is also possible that the interview questions that we put to the focus groups were too vague, and hence made it difficult for them to come up with examples.

### Implications for daily practice and further research

Several of the GPs experienced the focus group as an eye-opener. Potentially, the participants can take some of the insights from the discussion back to their own practice, and perhaps become emboldened to use communication techniques that they had abandoned or not previously mastered. This study might also imply that GPs need refreshing knowledge about patient-centered communication techniques that they have previously learned. Acknowledging patients’ strong sides can bolster GPs’ ability to help patients with medically unexplained symptoms. However, the epistemic disadvantage of generalist expertise makes this hard to achieve. It is difficult for GPs to integrate person-centered perspectives with biomedical knowledge due to the privileged position of the latter. This seems to indicate a need for system-level innovations to increase the status of person-centered clinical work.

Focusing on strengths may be useful not only when seeing patients with MUS, but for all patients living with chronic disease and therefore the findings may be applicable for GPs throughout much of their work. Our findings show that when discovering strengths in patients, the GPs felt they were able to provide better help when focus was changed from misery and negativity. This might also ultimately contribute to reduce the stigma and low status that this group of patients often experience.

While this study examined GPs’ experiences and reflections about becoming aware of patients’ strengths, it could be interesting to see a study on how patients experience consultations where the GP focuses on strengths.

## Conclusion

Acknowledging patients’ strengths holds a potential to bolster GPs’ ability to help patients with MUS. However, this requires a conscious effort on behalf of the GP.

## Supplementary Material

Supplemental Material

## Appendix 1.

### INTERVIEW GUIDE

1. Can you please share examples of having discovered strengths in patients with medically unexplained symptoms. What I am thinking about is something that can be considered to represent a strong side in the patient’s life outside the consultation room.
  - Why or how did the theme of the patient’s strengths come up during the consultation?
  - Can you please elaborate on the patient’s story / background?
  - Was it difficult to discover this strong side in the patient? What was the situation?
  - Did I understand it correctly when you said (…)?
  - Anyone else who has experienced something similar?
2. What significance did it have for you as a GP that you gained this knowledge about the patient?
  - Did anything change in your perception of the patient?
  - What did change?
  - What did you think when the patient started talking about this strong side?
  - Has anyone else had a similar experience?
  - How do you think this may have changed your relationship with this patient?
