# Supplementary material for: Discovering strengths in patients with medically unexplained symptoms – a focus group study with general practitioners
Source: Scand J Prim Health Care. 2022 Nov 8;40(3):405–13. doi: 10.1080/02813432.2022.2139345 (PMC9848323; doi:10.1080/02813432.2022.2139345)
Supplement: Supplemental Material [file IPRI_A_2139345_SM6247.docx]

**Appendix 1. INTERVIEW GUIDE**

1. Can you please share examples of having discovered strengths in patients with medically unexplained symptoms. What I am thinking about is something that can be considered to represent a strong side in the patient’s life outside the consultation room.

- Why or how did the theme of the patient’s strengths come up during the consultation?
- Can you please elaborate on the patient’s story / background?
- Was it difficult to discover this strong side in the patient? What was the situation?
- Did I understand it correctly when you said (…)?
- Anyone else who has experienced something similar?

2. What significance did it have for you as a GP that you gained this knowledge about the patient?

- Did anything change in your perception of the patient?
- What did change?
- What did you think when the patient started talking about this strong side?
- Has anyone else had a similar experience?
- How do you think this may have changed your relationship with this patient?
